# Re-Envisioning Anti-Apicomplexan Parasite Drug Discovery Approaches

**DOI:** 10.3389/fcimb.2021.691121

**Published:** 2021-06-11

**Authors:** Gabriel W. Rangel, Manuel Llinás

**Affiliations:** ^1^ Department of Biochemistry and Molecular Biology and the Huck Center for Malaria Research, Pennsylvania State University, University Park, PA, United States; ^2^ Department of Chemistry, Pennsylvania State University, University Park, PA, United States

**Keywords:** malaria, drug resistance, *Plasmodium*, antimalarial drugs, chemotherapy, screening

## Abstract

Parasites of the phylum Apicomplexa impact humans in nearly all parts of the world, causing diseases including to toxoplasmosis, cryptosporidiosis, babesiosis, and malaria. Apicomplexan parasites have complex life cycles comprised of one or more stages characterized by rapid replication and biomass amplification, which enables accelerated evolutionary adaptation to environmental changes, including to drug pressure. The emergence of drug resistant pathogens is a major looming and/or active threat for current frontline chemotherapies, especially for widely used antimalarial drugs. In fact, resistant parasites have been reported against all modern antimalarial drugs within 15 years of clinical introduction, including the current frontline artemisinin-based combination therapies. Chemotherapeutics are a major tool in the public health arsenal for combatting the onset and spread of apicomplexan diseases. All currently approved antimalarial drugs have been discovered either through chemical modification of natural products or through large-scale screening of chemical libraries for parasite death phenotypes, and so far, none have been developed through a gene-to-drug pipeline. However, the limited duration of efficacy of these drugs in the field underscores the need for new and innovative approaches to discover drugs that can counter rapid resistance evolution. This review details both historical and current antimalarial drug discovery approaches. We also highlight new strategies that may be employed to discover resistance-resistant drug targets and chemotherapies in order to circumvent the rapid evolution of resistance in apicomplexan parasites.

## Introduction

Apicomplexan parasites are prevalent all over the world and represent a major burden to global health, causing widespread diseases including cryptosporidiosis, one of the most common causes of diarrheal disease (Khalil et al., 2018), toxoplasmosis, infecting up to 30% of the global population (Montoya and Liesenfeld, 2004), and malaria, causing over 220 million clinical cases and approximately 400,000 deaths in 2019 (World Health Organization, 2019). Other Apicomplexan parasites like *Babesia* spp. and *Theileria* spp. massively impact agriculture and economic growth by infecting and killing livestock (Keroack et al., 2019). Thus, addressing the impact of these parasites is markedly important for human health and prosperity, and anti-parasitic chemotherapeutics are critical tools for treatment and elimination/eradication efforts.

Despite anti-apicomplexan research trailblazing drug development strategies for over 200 years, drug resistance has been a persistent challenge, with resistance to most major anti-apicomplexan chemotherapies reported in at least one targeted parasite species (Wongsrichanalai et al., 2002; McClure and Day, 2014; Montazeri et al., 2018). Given the current mortality rate of malaria and the potential for devastating increases in mortality in light of spreading *Plasmodium* spp. drug resistance, antimalarial drug resistance is a particularly pressing global issue (World Health Organization, 2019). Thus, while many of the points made herein are applicable to other apicomplexan parasites, the remainder of this review will focus on antimalarial drug discovery and will offer new approaches that may be adopted to identify putative resistance-resistant antimalarial drug targets.

## Early Antimalarial Treatment Discovery Approaches

Quinine, the earliest isolated compound used to treat malaria-induced fever, was first purified in 1820, 60 years before the discovery of the parasitic agent of malaria, from the bark of the South American cinchona trees, which was the key ingredient in a centuries-old traditional antimalarial remedy (**Table 1**) (Pelletier and Caventou, 1820; Achan et al., 2011). Subsequent attempts to abiotically synthesize quinine led to the 1856 discovery of aniline dyes (Brightman, 1956). By 1891, one of these dyes, methylene blue, was observed to selectively stain the recently discovered *Plasmodium* spp. parasites on malaria patient blood smears, and thus was clinically tested and found to be effective at clearing parasites from two patients (Ehrlich and Guttmann, 1891). While the dosage and side-effects were suboptimal, methylene blue denotes the birth of medicinal chemistry as it was a lead compound for future antimalarial development.

**Table 1:** Discovery methods of select antimalarial drugs.

Class	Drug	Compound Origin/ Screening Group	Species Initially Tested	Ref.
** **	**Quinine**	Extracts from the bark of the cinchona tree, an ancient South American antipyretic	Unspecified Human Malaria	(Pelletier and Caventou, 1820)
8-aminoquinolone Quinine Derivatives	**Methylene Blue**	Derivatives of aniline dye (initially synthesized trying to make synthetic quinine)	*P. falciparum* in humans	(Ehrlich and Guttmann, 1891)
	**Plasmochin**	Library of methylene blue derivatives	*P. relictum*	(Roehl, 1926)
	**Primaquine**	Library of methylene blue/plasmochin derivatives	*P. gallinaceum*	(Wiselogle, 1946)
	**Tafenoquine**	Library of primaquine derivatives	*P. falciparum and P. vivax in Aotus* monkeys	(Rossan, 1984)
4-aminoquinolone Quinine Derivatives	**Mepaquine**	Library of methylene blue derivatives	*P. relictum*	(Kikuth, 1932)
	**Chloroquine**	Library of methylene blue derivatives	*P. gallinaceum* and *P. lophurae*	(Loeb, 1946)
	Amodiaquine*	Library of chloroquine derivatives	*P. gallinaceum*	(Wiselogle, 1946)
	Mefloquine*	Library of quinine derivatives	*P. falciparum* and *P. vivax* in *Aotus* monkeys	(Schmidt, 1973)
	**Naphthoquine**	Library of quinine derivatives	*P. berghei*	(Li et al., 1982)
Other Quinine Derivatives	Piperaquine*	Library of quinine derivatives	*P. berghei*	(Benazet, 1965)
	**Pyronaridine**	Library of quinine derivatives	*P. berghei*	(Zheng et al., 1979)
	Lumefantrine*	Library of quinine derivatives	*P. berghei*	(Deng et al., 1997)
Antibacterial Drugs	**Sulfonamides**	Established antibacterial	*P. knowlesi*	(Coggeshall, 1938)
	**Tetracyclines**	Library of established antibacterials	*P. gallinaceum*	(Coatney and Greenberg, 1949)
	**Lincosamides**	Library of established antibacterials	*P. berghei*	(Lewis, 1967)
	Pyrimethamine*	Library of folate synthesis inhibitors	*P. gallinaceum* and *P berghei*	(Falco et al., 1951)
	**Azithromycin**	Library of erythromycin derivatives	*P. falciparum in vitro*	(Jensen and Gingras, 1992)
Other Drugs	Artemisinins**	Library of ancient Chinese medicinal plant extracts	*P. berghei*	(Su and Miller, 2015)
	**Halofantrine**	Library of highly aromatic compounds	*P. berghei* and *P. gallinaceum*	(Osdene et al., 1967)
	**Halofuginone**	Library of febrifugine derivatives	*P. falciparum in vitro*	(Geary et al., 1983)
	**Proguanil**	Library of non-quinoline/non-acridine ring structure analogs	*P. relictum* and *P. gallinaceum*	(Curd et al., 1945b)
	**Atovaquone**	Library of naphthoquinone derivatives	*P. berghei*	(Davies et al., 1989)

*Also used together with an artemisinin derivative as an artemisinin-based combination therapy (ACT).

**Includes artesunate, artemether and dihydroartemisinin.

## Animal Models for Antimalarial Discovery - Avians and Rodents and Monkeys, Oh My!

By 1921, canaries intravenously inoculated with blood-stage *P. relictum* were used as an animal model for malaria (Sergent and Sergent, 1921). This model was further developed by Bayer scientists to enable industrial-level screening of methylene blue analogs for antimalarial activity leading to the discovery of the first of the 8-aminoquinolone class of antimalarials, plasmochin (a.k.a. pamaquine, plasmoquine) (Roehl, 1926). Unfortunately, plasmochin also had severe side-effects, although screening of plasmochin analogs in *P. gallinaceum* for less toxic compounds eventually led to the discovery of primaquine, the most widely used 8-aminoquinolone (Wiselogle, 1946). Continued efforts to further increase tolerability and improve potency by the Walter Reed Army Institute for Research (WRAIR) led to the discovery of tafenoquine (a.k.a. WR 238605) *via* small-scale screening of primaquine analogs against *P. falciparum* and *P. vivax* in Aotus monkeys (Rossan, 1984).

Although 8-aminoquinolones were integral in malaria treatment, especially in preventing relapse of *P. vivax*, their poor efficacy against asexual blood stages was evident from the beginning. Thus, in parallel to 8-aminoquinolone development, the search for a partner schizonticidal antimalarial drug was undertaken at Bayer using *P. relictum* for screening of methylene blue analogs. These efforts resulted in the discovery of mepacrine (a.k.a. quinacrine, atebrin, atabrine, erion) (Kikuth, 1932), the first of the 4-aminoquinolones. Bayer continued iterative modification of the mepacrine structure and screening with avian malaria, discovering the most widely used 4-aminoquinolone, chloroquine (a.k.a. resochin, SN 7618) in 1934 (Loeb, 1946). Initially discarded, perhaps mistakenly, as too toxic, chloroquine was resurrected through the massive WWII antimalarial discovery efforts of the United States government, again, using an avian malaria model (Loeb, 1946; Wiselogle, 1946; Coatney, 1963). Amodiaquine (a.k.a. SN 10751, camoquin), a chloroquine analog was subsequently identified during the U.S. wartime efforts (Wiselogle, 1946), and mefloquine (a.k.a. WR 142490) emerged from small-scale screening of additional 4-aminoquinolone species against *P. falciparum* and *P. vivax* in owl monkeys in 1973 (Schmidt, 1973). Capitalizing on the observation that most previous successful antimalarials had high aromaticity, the antimalarial activity of halofantrine (a.k.a. WR 171669) was discovered, also by WRAIR, by screening multi-aromatic compounds against *P. berghei* (Colwell et al., 1972).

Starting in the 1930s, antibacterial agents were also explored for antimalarial treatment. By 1938, sulfanilamide, an early member of the sulfonamide drug class that includes sulfadoxine, was established as an antimalarial active against *P. knowlesi* in Rhesus macaques (Coggeshall, 1938). In 1949, aureomycin, a golden-hued chemical originally isolated from *Streptomyces* bacteria and the first antibiotic of the tetracycline class that includes doxycycline, was tested against *P. gallinaceum* and found to clear infections in chicks (Coatney and Greenberg, 1949). Lincomycin, the first of the lincosamide antibiotic class which includes clindamycin and pirlimycin, was isolated from *Streptomyces* from the soil in Lincoln, Nebraska (Macleod et al., 1964) and found to be effective against *P. berghei* (Lewis, 1967).

In the mid-1940s, while attempting to discover a better-tolerated schizonticidal drug, it became clear to the Bayer group that exploring other non-quinoline and non-acridine ring structure analogs might be more fruitful (Curd et al., 1945a). Through screening various cyclic chemotypes against avian malaria, proguanil (a.k.a. Paludrine, chlorguanide, chloroguanide), a pyrimidine analog, was discovered (Curd et al., 1945b). Around the same time, scientists at the Wellcome Research Laboratories in Tuckahoe, New York, initiated the first ever drug discovery approach targeting a specific metabolic pathway, by searching for purine and pyrimidine derivatives that inhibit *Lactobacillus casei* nucleic acid synthesis as measured by growth and acid production inhibition and verified by phenotype rescue with respective purine or pyrimidine media supplementation (Hitchings et al., 1950). Due to the similar activity of proguanil and 2:4-diaminopyrimidines in this new assay as well as their structural similarity, it was posited that compounds in this class might be antimalarial as well, and, indeed, screening of 2:4-diaminopyrimidine derivatives against *P. gallinaceum* and *P. berghei* led to the discovery of the antifolate pyrimethamine (a.k.a. No. 50-63, Daraprim) (Falco et al., 1951).

The current basis of first-line antimalarial combination therapies, artemisinin, was discovered through one arm of the massive Chinese military Project 523 focused on screening traditional medicines and folk remedies for antimalarial activity using the *P. berghei* model (Qinghaosu Antimalaria Coordinating Research Group, 1979; Su and Miller, 2015). The active compound was isolated in 1971 by professor and Nobel-laureate Youyou Tu from the *Artemisia annua* plant, which was a common ingredient in ancient Chinese antipyretic mixtures. Also through the extension of Project 523, the additional quinone variations, pyronaridine (a.k.a. 7351) (Zheng et al., 1979), lumefantrine (Deng et al., 1997), and naphthoquine (Li et al., 1982) were identified as antimalarial through initial screening against *P. berghei* (Cui and Su, 2009; Chen, 2014). Piperaquine (a.k.a. 13,228RP) was ultimately developed into a widely used antimalarial therapeutic in China (Chen, 2014) but was originally discovered as an antimalarial by the French through screening for long-lasting, synthetic antimalarials against *P. berghei* (Benazet, 1965).

## *In Vitro* Systems - Bringing Culture to Antimalarial Drug Discovery

In 1976, methods for the continuous *in vitro* cultivation of *P. falciparum* were published, simplifying antimalarial drug assays (Haynes et al., 1976; Trager and Jensen, 1976). Again, natural products served as a starting point when, as a part of the U.S. WWII wartime efforts, hydrolapachol was isolated from the bark of the lapacho tree, a traditional South American medicinal treatment, and through the avian malaria model, was discovered as the first lead antimalarial compound of the naphthoquinone chemical class (Wiselogle, 1946). Employing the *in vitro P. falciparum* culture system, the Wellcome Research Laboratories endeavored to improve upon hydrolapachol by screening naphthoquinone derivatives, resulting in the development of atovaquone (a.k.a. 566C80) (Davies et al., 1989).

During WWII, American scientists also isolated febrifugine, another lead antimalarial compound from a traditional Chinese antimalarial plant *Dichroa febrifuga* (Koepfli et al., 1947). While severe side effects paused development of this compound for human treatment, a number of derivatives including halofuginone (a.k.a. WR 273645) were commercially synthesized for use against coccidiosis of domesticated animals (Waletzky et al., 1967). Halofuginone was eventually confirmed as an effective antimalarial drug using the *in vitro P. falciparum* model (Geary et al., 1983; Jiang et al., 2005).

## Current Antimalarial Discovery Approaches - The Trouble With Resistance

Antimalarial resistance has been a major challenge since the early days of quinine, with evidence of reduced efficacy of every modern antimalarial treatment appearing within 15 years of widespread clinical introduction (Wongsrichanalai et al., 2002; McClure and Day, 2014). For example, once common antimalarial drugs such as chloroquine or pyrimethamine are often no longer widely recommended in typical malaria cases due to the rapid spread of parasites with mutant *pfcrt* or *pfdhfr* respectively (White, 2004). To slow the emergence of resistant parasites, the World Health Organization (WHO) has recommended that any novel antimalarial drug be clinically introduced in combination with a different partner antimalarial drug with a distinct mechanism of action (World Health Organization, 2006), which is why the newest frontline class of antimalarials, artemisinin and its derivatives, were rolled out globally in combination with various companion compounds as artemisinin-based combination therapies (ACTs). Unfortunately, even with ACTs there are increasing reports of delayed clearance of clinical parasitemias, most prominently in Southeast Asia but also now into the African continent (Amato et al., 2018; Uwimana et al., 2020). Rapid emergence of antimalarial drug resistance underscores the necessity to rethink antimalarial drug discovery approaches to minimize chances for resistance development and to maximize the longevity of novel drugs.

There are currently major efforts by international organizations such as the Medicines for Malaria Venture (MMV) and Malaria Drug Accelerator Consortium (MalDA) to identify and advance new antimalarial drugs and drug targets (MMV, 2020; Yang et al., 2021). Most modern antimalarial discovery approaches employ large-scale screening for *in vitro* parasite growth inhibition by compound libraries sourced from various commercial and academic groups such as GlaxoSmithKline (Gamo et al., 2010), St. Jude Children’s Research Hospital (Guiguemde et al., 2010), The Genomics Institute of the Novartis Research Foundation (Plouffe et al., 2008), Charles River Laboratories (Antonova-Koch et al., 2018), and The University of Dundee, Scotland (Abraham et al., 2020). From these and other libraries, MMV has collated compounds into open-access collections, such as the Pathogen Box or the Pandemic Box made of compounds active against a wide range of pathogens, or such as the Malaria Box, made with specifically antimalarial compounds, including both “drug-like” and “probe-like” structures that may serve as lead compounds for future chemotherapeutics or research reagents respectively (Spangenberg et al., 2013; MMV, 2021; Samby et al., 2021).

Subsequent to initial screens, studies aim to define mechanisms of action of identified lead compounds before further development in animal models (Cowell and Winzeler, 2019). *Plasmodium* experimentation has been facilitated greatly by the application of modern tools such as transposon screening to identify essential genes (Zhang et al., 2018), CRISPR-Cas9 for rapid DNA modification (Lee et al., 2019), and additional “-omics” technologies (Cowell and Winzeler, 2019). Unfortunately, many of the further analyzed hit compounds often share common resistance mechanisms, such as through mutations in *pfatp4*, *pfcytb*, or *pfdhodh*, rendering the large amount of resources put into discovery and characterization vulnerable to undesirable cross-resistance issues (Guiguemde et al., 2010; Spillman and Kirk, 2015).

A minor subset of modern antimalarial drug discovery endeavors have been target-based, seeking inhibitors of specific parasite enzymes such as PfCDPK1 and PfPI4K (Kato et al., 2008; McNamara et al., 2013). However, there is no reason to believe this approach will result in slower resistance development. In fact, the leading method currently used to identify resistance mechanisms is *in vitro* evolution and whole-genome analysis (IVIEWGA), which inherently demonstrates the relative ease with which the parasites can adapt to overcome the pressure from these lead compounds (Cowell and Winzeler, 2019; Winzeler, 2019). There are also cases when *in vitro* evolution fails, leading researchers to label these as “irresistible” or “resistance-refractory” compounds, which are particularly promising as lead molecules for resistance-resistant drug development (Vanaerschot et al., 2020; Yang et al., 2021). However, because irresistible compounds are the minority of compounds analyzed by the IVIEWGA method (Luth et al., 2018), which is labor and resource intensive, and because discovering the irresistible characteristic of these compounds comes only subsequent to initial investments of large-scale screening and winnowing of candidates, this approach is inefficient in discovering promising resistance-resistant antimalarial chemotherapies. Thus, there is a need to reevaluate antimalarial drug discovery approaches, focusing on designing strategies to discover resistance-resistant targets and compounds.

## Potential Resistance-Resistant Antimalarial Drug Discovery Approaches - Taking Notes From Other Fields

The idea of resistance-resistant drug discovery has been considered in other fields from anti-cancer therapies to antibiotic development. One appealing approach is to target novel vulnerabilities developed as a pathogen or cancer cell subsequently adapts to becoming drug resistant. This idea of “collateral sensitivity” was originally discussed in 1952 when it was discovered that various strains of *Escherichia coli* resistant to different antibiotics became more sensitive than their parental strain to other antibiotics (Szybalski and Bryson, 1952). Identifying and targeting collateral sensitivities has been successfully applied *in vitro* to cancerous cells (Pluchino et al., 2012), clinical bacterial isolates (Imamovic and Sommer, 2013), and even *P. falciparum* on a limited scale (Lukens et al., 2014; Ross et al., 2018). These *Plasmodium* studies screened chemical libraries for compounds that would selectively kill drug-resistant parasite cultures or inhibit drug-resistant *Plasmodium* enzymes in *in vitro* biochemical assays. Both studies found targeting collateral sensitives in *P. falciparum* to be promising. To our knowledge, this technique has not yet been applied in clinics, likely because these studies are relatively nascent, but it does have potential to revolutionize chemotherapeutic approaches aimed at avoiding resistance development. For malaria treatment, adherent to the WHO recommendation that all new antimalarial therapies be combinations of multiple drugs, designing the combination therapy with a primary drug and a secondary drug that targets a collateral sensitivity could potentially lock parasites in a sensitive state, significantly increasing the effective longevity of novel treatments.

While strategies to identify vulnerabilities in drug resistant cell lines exist, their limitations necessitate the consideration of novel approaches. The most widely implemented tactic to discover collateral sensitivities has typically been to screen resistant cells lines against existing lead compounds or drugs. Current capabilities for large-scale screening of compounds for antimalarial activity could easily be adapted to this approach by simply changing the parasite line used (Yang et al., 2021). However, the well documented diversity in evolutionary paths to resistance means collateral sensitivities are often not reproducible in similar resistant cell lines from other experiments, lineages or patients (Nichol et al., 2019). Alternatively, wholistically charactering the biological pathway adaptations acquired by resistant cell lines, through identifying genomic, transcriptomic, and/or metabolomic alterations in resistant lines, may enable the identification of entire collaterally sensitive biochemical pathways. Unlike discovering collateral sensitivity to a specific compound, which likely corresponds to only one collaterally sensitive target, understanding new pathway vulnerabilities may elucidate many putative drug targets.

Finally, another resistance-resistant antimalarial drug discovery approach capitalizes on the inherent parasitic nature of the malaria pathogen. Throughout its life cycle, the parasite is highly dependent on resources from the host environments including the human liver and blood, and the mosquito midgut and salivary glands. For instance, blood-stage *Plasmodium* parasites are known to rely on many host sources including hexose, glutamine, fatty acids and purines (Olszewski and Llinas, 2011; Cheviet et al., 2019). Chemotherapeutically targeting host processes is routinely used in clinical treatments for bacterial and viral infections (Zumla et al., 2016). While most host-targeted treatments mitigate inflammatory responses, host metabolism can also be targeted, although this is often challenging since treatments must be carefully designed and managed to ensure that vital host metabolism remains sufficient. Nevertheless, it has found clinical success as is the case with inhibiting host cholesterol biosynthesis to reduce dengue virus replication (Rothwell et al., 2009). For *P. falciparum*, there is strong evidence that the host heme biosynthesis enzyme, ferrochelatase, is required for normal parasite blood-stage growth, and given that up to 70% inhibition of ferrochelatase activity is relatively well tolerated as in patients with natural polymorphisms, this enzyme has been proposed as a host-directed chemotherapeutic target (Smith et al., 2015). A more complete characterization of the parasite dependencies on host functions could elucidate novel host-directed antimalarial drug targets over which the parasite has no evolutionary control, thereby rendering it less susceptible to resistance.

## Conclusion

Malaria research has propelled innovation in drug discovery for over two centuries. Since the purification of quinine in 1820, antimalarial drug research has had a rich history, including many advances applicable not only to malaria but drug development in general. Notably, with quinine, the malaria field is responsible for the first ever purification of a clinically active ingredient from natural products. Subsequently, antimalarial discovery efforts brought forth the age of medicinal chemistry and established the roots for some of the largest and still relevant pharmaceutical companies in history. Additionally, malaria served as the catalyst for initiating multiple military-led, massive drug discovery efforts from which many current drugs for malaria and other diseases were developed.

Recently, the efforts of organizations such as MMV, the MalDA consortium and other individual teams have stocked the current antimalarial pipeline with promising drugs in various stages of development. Unfortunately, malaria researchers, clinicians and patients continue to face the ever-present challenge of drug resistance. Furthermore, the enormous progress the world has made in reducing malaria morbidity and mortality over the last two decades has stalled in recent years. Therefore, now is the time for the malaria research community to critically and creatively consider alternative approaches to antimalarial drug discovery. Future efforts should include an emphasis on developing resistance-resistant therapies and can do so by incorporating successful ideas evaluated by other fields and by innovating new strategies. If we are successful in establishing robust resistance-resistant drug discovery strategies for malaria, we will be that much closer to eradicating this disease and could once again revolutionize the global field of drug discovery.

## Author Contributions

GR and ML performed all literature review, manuscript drafting and table drafting. All authors contributed to the article and approved the submitted version.

## Funding

GR is supported by the Pennsylvania State University Eberly College Postdoctoral Research Fellowship. ML gratefully acknowledges support from the Bill and Melinda Gates Foundation (OPP1054480).

## Conflict of Interest

The authors declare that the research was conducted in the absence of any commercial or financial relationships that could be construed as a potential conflict of interest.
